# Ultraslow spreading ridges: slowest but locally thickest

**DOI:** 10.1093/nsr/nwae399

**Published:** 2024-11-07

**Authors:** Chuan-Zhou Liu, Ri-Xiang Zhu

**Affiliations:** Laoshan Laboratory, China; State Key Laboratory of Lithospheric and Environmental Coevolution, Institute of Geology and Geophysics, Chinese Academy of Sciences, China; State Key Laboratory of Lithospheric and Environmental Coevolution, Institute of Geology and Geophysics, Chinese Academy of Sciences, China

The mid-ocean ridge (MOR) is the longest volcanic chain on the Earth, with a total length of ∼75 000 km and spreading rate varying from fast (>80 mm/yr) to ultraslow (<20 mm/yr). At the MOR, divergence leads to decompressional melting of the asthenosphere, producing melts for crustal accretion. Geophysical observations on global ocean ridges have revealed that seismic crustal thickness shows little dependency on spreading rate until it is <20 mm/yr, with a global average of 6 ± 1 km. Such a constancy in thickness has been perfectly explained by a passive flow model, in which the hot buoyant asthenosphere rises passively to the melting zone.

At ultraslow spreading ridges, enhanced conductive cooling and hydrothermal circulation thicken the ocean lithosphere, which shrinks the melting zone and inhibits melting. These effects are combined to subdue magma supply and generate thin oceanic crust. Attenuated crustal thickness under ultraslow spreading conditions is well predicted by the passive flow model and also justified by geophysical observations. Nevertheless, anomalously thickened crust up to 10 km has been discovered at the ultraslow Southwest Indian Ridge (SWIR), challenging the validity of the passive flow model [1]. However, deciphering the mechanism for the overthickened ocean crust at SWIR is unavoidably entangled with other factors, such as oblique spreading, mantle plumes and hotspots. Therefore, why overthickened crust occurs at the ultraslow spreading ridges, and the underlying fundamental dynamics governing crustal accretion, remain unknown.

The Gakkel Ridge in the Arctic Ocean, with a full spreading rate of 7–14 mm/yr, is ideal for studying crustal accretion under ultraslow spreading conditions, as it is circumvented by other factors like oblique spreading, large-offset transform faults or nearby hotspots. However, the perennial cover of sea ice makes the Gakkel Ridge the hardest place for geological and geophysical surveys. Seismic exploration using conventional ocean bottom seismometers (OBSs) has been deemed almost impossible [2]. During the Arctic Mid-Ocean Ridge Expedition (AMORE) in 2001, two ice-breakers, *Polarstern* and *Healy*, were jointly deployed and a few ice-station-based seismic investigations were conducted [3]. The results have revealed the occurrence of ancient mantle domains up to 2 billion years [4] and exceptionally thin crust (i.e. 1.9–3.3 km) along the Gakkel Ridge [2].

**Figure 1.**
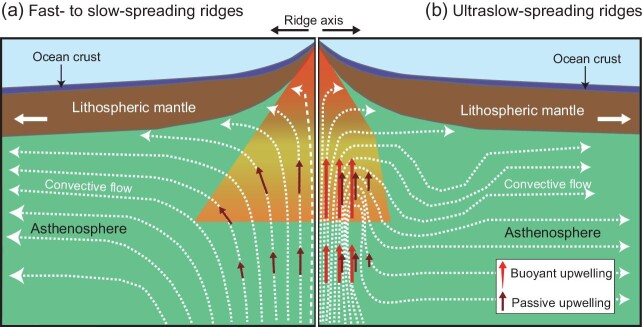
Cartoon of mantle dynamics beneath mid-ocean ridges. (a) At fast- to slow-spreading ridges, the buoyant asthenosphere upwells passively and gives rise to basaltic magmas once it enters the melting zone. (b) At ultraslow spreading ridges, the active upwelling of the asthenosphere predominates over the passive upwelling, probably because its buoyancy and viscosity have been enhanced by entrapped melts.

In 2021, the Joint Arctic Scientific Mid-ocean ridge Insight Expedition (JASMInE) led by Chinese scientists took on the impossible and, for the first time, conducted a high-resolution seismic survey using an OBS array at the eastern segment of the Gakkel Ridge [5]. Unlike the strongly attenuated crust obtained previously [2], the OBS data unexpectedly revealed that the along-axis crustal thickness of its eastern segment is comparable with that of global ocean ridges. In particular, an overthickened crust up to 8.9 km (with an uncertainty of 0.1–0.5 km) has been discovered on-axis at 100^o^E. After ruling out other possibilities, the authors attributed the thick crust to the dominant role played by active upwelling over the passive flow of the asthenosphere beneath this region (Fig. 1). Although the fabulous success achieved by JASMInE changes tremendously our knowledge of mantle dynamics at spreading centers, some important issues still remain unresolved. For instance, what is the depth and morphology of the lithosphere–asthenosphere boundary (LAB) beneath this region? How do melts migrate and aggregate beneath the ridge axis?

The Arctic Ocean has become a hot topic for international science communities. Besides the Gakkel Ridge, the ecosystem of the Arctic Ocean has also attracted a lot of attention. To embrace the challenges and international competition, a Chinese team led by Prof. Jiabiao Li has successfully applied for a project, Arctic Deep Observation for Multi-sphere Cycling (ADOMIC, 2022–2030), hosted by the United Nations Decade of Ocean Science for Sustainable Development Programme. This project focuses on the ecosystem of the Arctic Ocean and initiates multidisciplinary and multiscale research on the multi-sphere (including lithosphere, cryosphere, hydrosphere and biosphere) mass and energy exchange in this region. Implementation of the ADOMIC Project heralds a new era of multidisciplinary studies on the Arctic Ocean.
